# Veratrum Viride

**Published:** 1856-12

**Authors:** R. E. Haughton


### VERATRUM VIRIDE.

BY R. E. HAUGHTON, M. D.

PROFS. BOWLING AND EVE:—Gentlemen, enclosed I review some ideas advanced by Dr. Brown, on the uses and effects of Veratrum Viride.

I propose to offer some views demonstrated to me in practice, and afterwards to notice the article before mentioned. This remedy was more fully brought before the profession as Norwood's Tincture. This tincture is made from the root of the American hellebore, an undigenous plant, and possesses some very remarkable and powerful therapeutic properties, which renders it one of our most valuable medicinal agents in many forms of disease. Dr. Norwood gives to it not less than eight distinct properties, or therapeutic effects. Its sedative properties are probably its most important, though possessing other very valuable remedial effects. It is first sedative, nauseant, diaphoretic, and a powerful nervine, modifying exalted nervous sensibility, restlessness, more promptly than most other articles of that class.

We propose to consider its properties first then as a sedative, and give some reasons, drawn from experience, why we regard it as among the most reliable and valuable articles of the materia medica. It may be confidently relied upon to control the action of the heart and arteries in all febrile and inflammatory diseases, reducing the pulse from 140 beats to 60, or even lower, in a period of time ranging from six to twenty-four hours, and which may be maintained so long as desirable, thus being a great desideratum sought by the medical world. In all idiopathic fevers the first lesion is found in the nervous system and brain, and this influence operating upon the sanguiferous system produces excitement, general fever, and the first and most prominent indication is to modify this excitement. Why? Because this increased excitement produces congestion, irregular determinations of blood, and the chain of morbid action goes on lengthening till serious complications may exist. The veratrum viride then fully meets the indication controlling the heart's action, and keeping up a normal circulation. In the phlegmasia the result is the same, curing inflammation by removing the cause, increased circulation; first, causing local congestion, then inflammation. But how does this cure inflammation? it may be asked. First, what is inflammation? Williams in his pathology defines it thus, "Motion partly increased, and partly diminished in the capillary circulation of the part laboring under the morbid action. Motion increased outside of the point where actual inflammation exists, and decreased within the point of attack." According to the physiological doctrine, (*ubi irritatio ibi fluxus*,) a flow of blood takes place to the point of inflammation, and especially is this so when the arterial action is increased still more by the force of the local trouble. The heart by its rapid action is constantly injecting the vessels of the part involved in trouble, and thus extension of the inflammation takes place, till the whole structure of an important organ may be passing through the different stages of progress, which is to end in disorganization and death. In place then of venesection, the use of this sedative comes to our aid as a more reliable means of medication, and more fully demonstrating the motto, *contraria contrarius curantur*. And again, this article can be used when depletion would be contra-indicated by the debility of the patient. My own experience teaches me, that in low conditions, when the heart's action is very feeble and rapid, this remedy may be used, reducing the number of beats, and as this is done, the pulse becomes fuller and softer, while the extremities, which had been cold from deficient circulation, now become warm, and the shrunken skin again resuming its natural color, fulness, etc. The sedative, then, does not weaken the powers of life under such circumstances, but acts as a conservator of the life forces. These points I have witnessed at the bed-side, when hope, which had almost gone glimmering among the things of the past, revived again, and bid us be of good cheer. Life is safe. I have thus administered in typhoid fever, inflammation of the lungs, brain, and I have not done as some men do, prescribe a dozen different remedies along with some special one, and then attribute all the good results to one alone. This is false experience. I have obtained these results when the system had received no other remedial impression. I rely upon this medicine in the treatment of typhoid fever more than any other one article, yet when the indications require, I do not reject others, and this is the experience of many of my professional friends who have tried it at my request.

But now we come to Brown on tincture of veratrum viride. Is it an abortive? And in any case, is it safe? Dr. Brown takes the position that it is an abortive, and that it is a dangerous remedy in any case. Dr. Newsom has used this remedy, it seems, not only in cases of pregnancy, with the happiest effects, but in many cases of inflammation, without any ill effects. Well, now hear what is thought of this opinion. Dr. Brown supposes that Dr. Newsom gave it and shot for his office, and says, "If he can give it in sedative doses to three persons, and carefully watch it and see no ill effects in at least one, he can do what the Doctors in this beat have all failed to do." Well, now I shall have to add my experience to that of Dr. Newsom. Having used this remedy freely but carefully for the last three years, and in cases of pregnancy in all its periods, having had no case of abortion, either from that or any thing else, during the time of using it. Further, I have used it in many other forms of disease, in all ages from the infant to adult age, and have never had any untoward symptom in any case, and have always attained the desired end. Again, the position of Dr. Brown in regard to its being an abortive is wrong in theory, and must fail in practice. There is no effect ascribed to it by writers heretofore which could produce this effect, except its nauseant or emetic effect. And will not ipecac, lobelia, antimony, or any of this class produce abortion if administered to their full effect. But the full effect desired from veratrum viride is produced ere emesis or even nausea manifests itself. Then it must have been an abuse of the remedy or improperly administered. Then let not our friend try to obscure his practice by charging effects upon this remedy which it does not possess, and thereby injure the remedial standing of a valuable article. Thus have many of our most reliable agents become depreciated in value, while a more careful experience would have placed them in estimation, where they properly belong.

Again, there are many diseases, which when they attack the pregnant female will, sooner or later in their course, produce abortion. All fevers and forms of inflammation have this tendency. Now is it possible Dr. Brown has overlooked this matter, and in such cases attributed abortion to the effects of tincture veratrum viride, when in truth it was the effect of a general disease reflecting its influence upon the gravid uterus. Now the generally received opinion is, that any remedy which acts as a sedative is an anti-abortive. For instance some practitioners would bleed in a case of threatened abortion, if there was much arterial excitement. Why? Because it would modify the force of arterial action. Now upon what principle of pathology is this based? First, arterial excitement acts upon the circulation of the gravid womb, and by the force thus thrown through arterial branches, detaches the placental from the uterine surface, causing hemorrhage, and this effusion of blood continues till the placenta is separated, and then it is in all respects a foreign body, and uterine contraction disposes of it together with the germ of humanity, which was being developed. Now if the effect of a good sedative can early be obtained, this process is arrested mainly through the control of the arterial system, and if any small amount of interstitial hemorrhage took place, absorption takes place and the disruption is healed, and development of the embryo goes on. Now this view is based upon a principle too evident to admit of doubt. Yet we are told this sedative is an abortive. The same principle is involved in any case of hemorrhage sustained and fostered by increased arterial action, thus it is that veratrum viride becomes a good remedy in such cases, giving a chance by the same control which it exerts here for bleeding vessels to be closed by nature's effort—coagulation. Again, I have used this remedy in epilepsy and with satisfactory results. One case of 10 year's standing has now been clear of an epilectic seizure for 12 months from date of first administration, and it produced a sedative action fully in this case. Thus I might go on enumerating cases, demonstrating its efficiency and safety, but for the present I forbear.

I think I have shown clearly that I am borne out in the careful and judicious use of this remedy, and that the effects attributed to it do not obtain as a rule, but if at all they are the exception, as I have shown such influences might come from some of our most common remedial agents.

